# Effects of dairy product intake and home-based resistance exercise on the physical function in Japanese community-dwelling older adults: results of a real-world randomized controlled trial

**DOI:** 10.1186/s12877-026-08061-x

**Published:** 2026-08-04

**Authors:** Ryo Miyazaki, Naoki Sakane, Hitoshi Ando, Makoto Ayabe, Hirokazu Taniguchi, Masayuki Yamasaki

**Affiliations:** 1 Faculty of Human Sciences, Shimane University, 1060 Nishikawatsu, Matsue, Shimane 690-8504 Japan; 2 Division of Preventive Medicine, Clinical Research Institute for Endocrine and Metabolic Disease, National Hospital Organization, Kyoto Medical Center, Kyoto, Japan; 3 Department of Cellular and Molecular Function Analysis, Kanazawa University Graduate School of Medical Sciences, Ishikawa, Kanazawa Japan; 4 Faculty of Computer Science and Systems Engineering, Okayama Prefectural University, 111 Kuboki, Soja, Okayama, 719-1197 Japan; 5 Department of Agriculture, Ryukoku University, 1-5 Yokotani, Seta Oe-cho, Otsu, Shiga 520-2194 Japan

**Keywords:** Aging, Elderly, Milk, Muscle strength, Nutrition

## Abstract

**Purpose:**

Although the combination of dairy product intake and resistance training has shown benefits for muscle health in older adults, most intervention studies have been conducted under highly controlled conditions, limiting their applicability to real-world settings.

**Methods:**

We conducted a 12-week pragmatic, open-label randomized controlled trial involving community-dwelling Japanese older adults. Fifty-four adults aged ≥ 65 years were assigned to either a dairy product intake plus home-based resistance training group (milk and exercise; ME) *or a* home-based resistance training only group (E). The dairy intervention targeted an increase equivalent to approximately 10–15 g of *dairy-derived protein* per day within habitual meals. Both groups were instructed to perform home-based resistance training at least three days per week. The physical function, including upper and lower limb strength, handgrip strength, gait speed, and body composition, was assessed before and after the intervention. Exploratory subgroup analyses were conducted according to baseline dairy consumption and skeletal muscle mass.

**Results:**

Fifty-one participants (mean age, 78.2 ± 6.9 years; BMI, 22.9 ± 3.2 kg/m²) completed the study (retention rate: 94.4%). Adherence to milk supplement consumption was exceptionally high (median 7.00 times/week, IQR 7.00–7.00 times/week). The median frequencies of milk and dairy product intakes were 6.83 (IQR 5.60–7.00) and 6.83 (IQR 6.17–6.94) times/week, respectively. Resistance exercise adherence was comparable between the ME and E groups (3.13 [2.81–3.65] vs. 3.25 [2.71–3.96] times/week; *p* = 0.940). No significant differences were observed between the groups in terms of muscle strength, physical function, or body composition. However, some functional outcomes improved over time in both groups. In exploratory subgroup analyses, participants with lower habitual dairy consumption at baseline showed improvements in upper and lower limb strength only in the ME group.

**Conclusions:**

Adding modest dairy product intake to home-based resistance training did not provide additional overall benefits compared to exercise alone. However, exploratory findings suggest that baseline dairy consumption may influence the responsiveness of older adults to combined nutrition and exercise interventions.

**Trial registration:**

Registration number UMIN000052127 Date of registration 2023/09/08 URL https://center6.umin.ac.jp/cgi-open-bin/ctr/ctr_view.cgi?recptno=R000059497.

**Supplementary Information:**

The online version contains supplementary material available at https://doi.org/10.1186/s12877-026-08061-x.

## Introduction

Milk and dairy products are rich sources of several essential nutrients, including high-quality proteins, bioactive lipids and fatty acids, minerals, and vitamins [1], and have been shown to help maintain and increase skeletal health in older adults [2]. Several studies suggest that combining milk-derived proteins with resistance training enhances musculoskeletal outcomes beyond either strategy alone in older adults [3] or those with sarcopenia [4]. However, evidence remains inconsistent for functional endpoints, such as muscle strength and performance, with some studies reporting no effects despite increases in lean mass [3, 5]. Similarly, nutritional guidance recommends distributing approximately 25–30 g of high-quality protein per meal to stimulate muscle protein synthesis in older adults [6], and approximately 80% of older adults fail to meet this recommended intake level in the general population [7].

The important limitations of previous intervention studies seem to be the reliance on supervised training, higher exercise volumes, and milk-derived protein doses, which may be difficult to sustain outside research settings. A lower training volume has been implicated in attenuating hypertrophic responses in community-dwelling older adults [8], and pragmatic studies combining dairy products and resistance training have reported limited functional gains, potentially due to participant heterogeneity (e.g., habitual dairy consumption and muscle health such as with/without sarcopenia) and issues of low adherence [1, 9]. Although long-term trials indicate that protein supplementation combined with structured resistance training can preserve muscle mass and improve strength under limited conditions (heavy resistance training) [9], many older adults prefer home‑based, low-intensity, low‑cost activities that are more accessible and acceptable [10]. Therefore, there is a need for pragmatic randomized trials that evaluate modest, affordable increases in dairy intake, together with unsupervised, home-based resistance training under “real-world” conditions.

Emerging evidence also suggests that baseline characteristics among older adults may modify their responsiveness to nutrition/exercise interventions. Gains in muscle outcomes can be blunted when habitual protein intake is adequate [11, 12], whereas individuals with lower baseline nutrient intake (such as low calcium intake) or poorer lifestyle profiles (such as sedentary lifestyle) are more likely to benefit from health outcomes [13]. An epidemiological study has suggested potential thresholds for dairy intake levels, such as ≥ 1 [14] and ≥ 2.2 [2] servings/day, related to functional phenotypes in older adults. In addition, a previous study [15] found that after 16-week resistance training, older adults with lower baseline fitness levels, including muscle mass, showed greater improvements in muscle health in comparison to fitter individuals. Taken together, from a clinical perspective, evidence is lacking on whether habitual dairy consumption and baseline muscle mass can modify the effects of pragmatic home-based dairy product interventions on functional outcomes.

Therefore, the present study aimed to investigate whether increasing the intake of dairy products containing dairy-derived protein, combined with home-based resistance training, can improve physical function relative to home-based resistance training alone. The primary outcomes were upper- and lower-limb muscle strength, and the secondary outcomes included handgrip strength, gait speed, and body composition. Given the plausibility of effect modification, we further conducted pre-specified exploratory subgroup analyses based on habitual dairy intake and muscle mass at baseline.

## Methods

### Study design (Fig. 1)

**Fig. 1:**
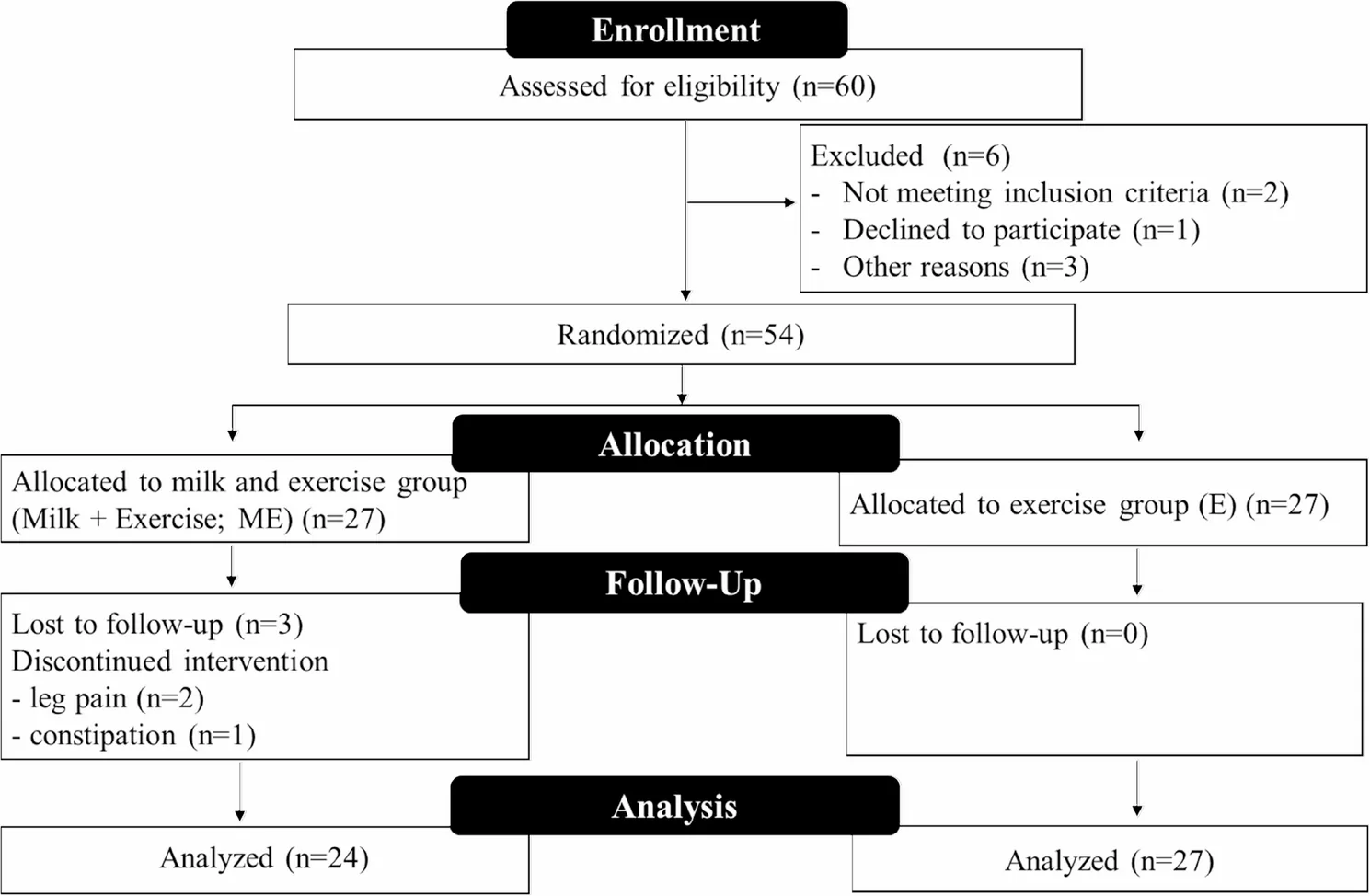
CONSORT flow diagram of participant recruitment, randomization, follow‑up, and analysis

This 12 week, parallel-group, open-label, single-center, randomized controlled trial aimed to test whether modest daily dairy product intake (targeting approximately an increase in dairy products corresponding to approximately 10–15 g/day of dairy-derived protein within routine meals) combined with home-based resistance training (≥ 3 days/week) improves physical function relative to home-based resistance training alone. This trial was conducted under real-world conditions and is reported in accordance with the Consolidated Standards of Reporting Trials (CONSORT) guidelines. Eligible participants were randomly assigned to the milk and exercise group (milk and exercise; ME group: combination of dairy product intake and home-based resistance training) or the exercise group (E group: resistance training only) in a 1:1 ratio, based on pre-measurement dairy consumption and muscle mass, as described below. Both groups were assessed before and after the 12-week study period (Fig. 1).

### Participants

Community-dwelling older adults were recruited using fliers displayed in Matsue City (Shimane, Japan). All participants were free from severe cardiovascular disease according to their medical history and lived independently. All participants provided written informed consent, and the ethics committee of the Medical School of Shimane University approved this study (7103). The inclusion criteria were: (1) age ≥ 65 years and (2) consent to participate in the present study. The exclusion criteria were (1) reported milk allergy or lactose intolerance (abdominal pain or diarrhea), (2) individuals for whom exercise was contraindicated by their family physician, (3) resting systolic blood pressure (SBP) ≥ 180 mmHg [16], and (4) difficulty in exercising the lower extremities (knee extension and bending).

### Randomization

The groups were blocked, stratified randomized, and assigned based on the pre-measurement dairy consumption and muscle mass. Briefly, the frequency of dairy consumption (≥ 1 cup/day or < 1 cup/day) was determined using a self-administered questionnaire according to the Japanese Food Guide Spinning Top [17]. The randomization process was managed and operated by an independent third party.

### Sample size calculation

The minimum sample size was determined based on the muscle mass in our study. Although the primary outcomes were upper- and lower-limb muscle strength in the present study, the sample size was calculated based on muscle mass because no published data were available for these specific strengths. The required sample size for the definitive trial was extrapolated from a prior study that assessed the combination of dairy product intake and resistance training in older adults [18]. G*Power (ver. 3.1.9.2; Universität Düsseldorf) was used, utilizing Cohen’s effect size of 0.85, α of 0.05, and power of 0.8, the estimated sample size was 46. To account for a potential 10% dropout rate [8], the final sample size was 52 participants (26 in each group).

### Intervention

This three-month intervention consisted of home-based resistance training (both ME and E groups) and milk dairy product intake (ME group only).

### Home-based resistance training (Fig. 2)

**Fig. 2:**
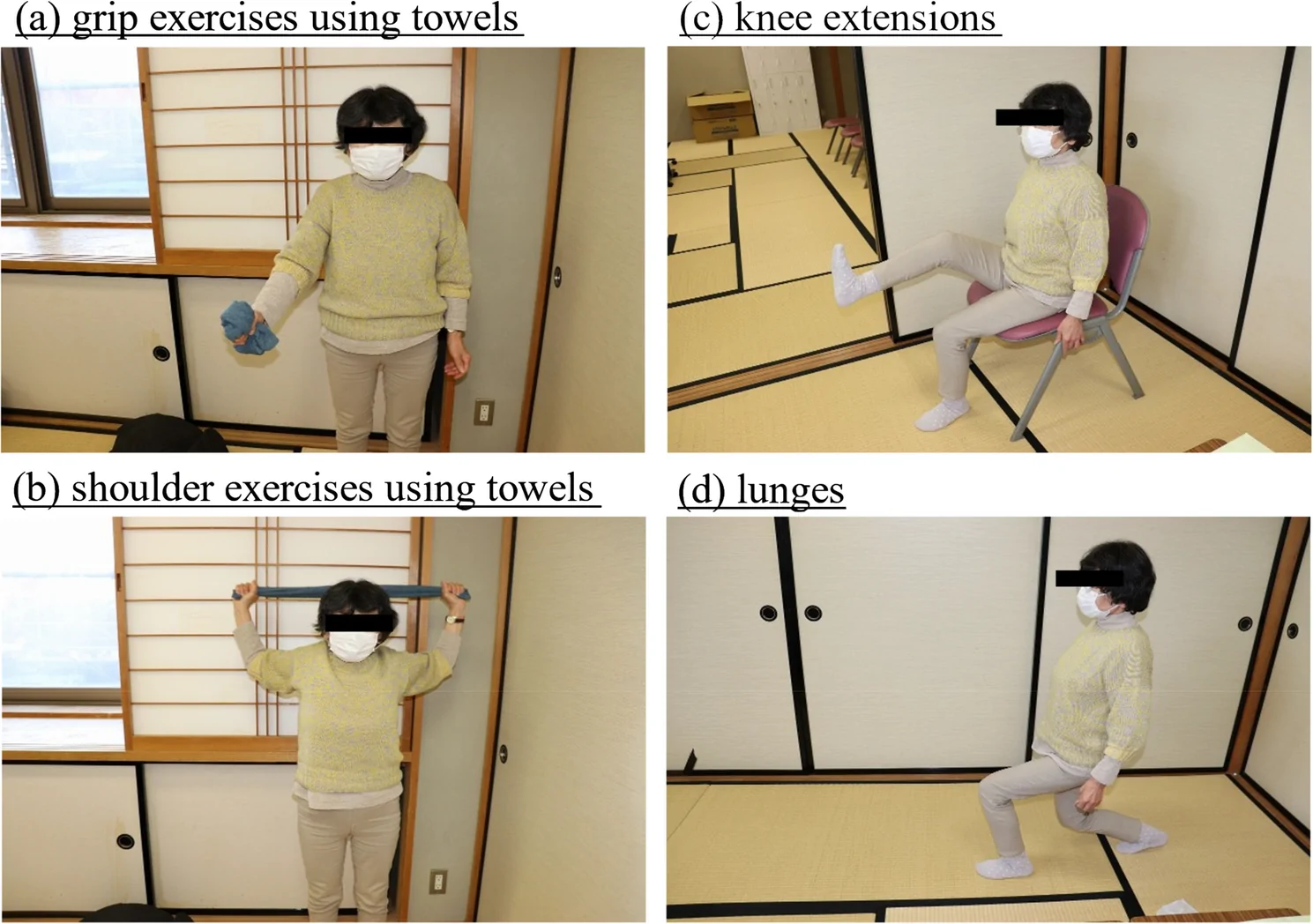
Home-based resistance training. (**a**) grip exercises using towels; (**b**) shoulder exercises using towels; (**c**) knee extensions; (**d**) lunges

Both groups (ME and E groups) were instructed to perform the same home-based exercise menu, mainly resistance exercises (calisthenic training using their own body weight), at least three times a week. Participants were asked to complete all four exercises in a single session. The prescribed resistance training (four exercises) was designed to be performed at home without equipment, targeting the lower and upper body. Upper-extremity training included shoulder and grip exercises using towels, while lower-extremity training included knee extensions and lunges. Participants performed each exercise for 5–15 repetitions per set for two sets, with the number of repetitions adjusted according to individual ability (Fig. 2). The exercise load was determined based on previous studies [19], a setting of approximately 5–7 (somewhat hard) on the OMNI-RES (0–10) [20]. Note that the setting of 5–7 on OMNI-RES is equivalent to 50%–75% of the one repetition maximum (1RM) [20]. Before the intervention, each exercise load (reps) was determined by trained exercise trainers using the pre-measurement data.

Upper-extremity training consisted of towel-based exercises. Shoulder exercises were performed by moving the arms while holding a towel to provide light resistance to the shoulder muscles. Grip exercises using towels were performed by repeatedly squeezing or grasping a towel to train handgrip strength. Lower-extremity training consisted of knee extensions and lunges targeting the major muscles of the lower limbs. Knee extensions were performed as seated lower-limb exercises to train the knee extensor muscles. Lunges were performed as standing lower-limb exercises to strengthen the thigh and hip muscles.

They could perform the exercises at any time of the day. Exercise adherence was monitored using monthly logs. A weekly community health class was available but did not include structured exercise instruction related to the study protocol; attendance was optional and was not considered part of the intervention. Therefore, the intervention was operationally defined as home-based.

### Dairy product intake

Participants randomized to the ME group were instructed to increase their consumption of dairy products, including milk, yogurt, cheese, and a milk-based supplement, with a target increase of approximately 10–15 g/day of dairy-derived protein. Before the intervention, trained nutritionists explained to each participant which foods contained milk-derived protein and in what amounts, and advised them on how to easily consume it. The participants were provided with a comprehensive list of dairy products prepared by the registered dietitians. No specific timing or food items were designated, allowing participants to select the timing and products that best suited their daily routines. Although the choice of dairy products and intake methods was left to the participants’ discretion, they were requested to avoid making major changes to their dietary habits during the study period. Considering that some participants do not typically consume much dairy, a milk supplement (“Milk Seikatsu Plus Stick Type”; 3.8 g protein/stick) was provided to all participants. Intake was monitored using daily self‑reported logs, in which participants recorded the type of dairy products consumed and whether they consumed the dairy products. While these logs allowed for tracking the frequency and compliance of daily consumption, the exact portions or weights (g/day) of routine meals were not directly measured. Participants in group E received no dietary instructions.

### Measures

Before and after the intervention, all participants underwent measurements after a 12-h fast to standardize hydration status and metabolic conditions across participants [21] at Shimane University.

### Muscle strength (upper and lower limb strength, handgrip strength)

The primary outcome measure was the physical function. Upper and lower limb strength included an arm curl test (functional upper limb strength indicator) [22] and a 30-second chair-stand test (functional lower limb strength indicator) [23]. For the arm curl test [20], the participants lifted a dumbbell (five pounds for women and eight pounds for men) on the self-reported dominant side while sitting on an armless chair. The tester stood next to the participant, holding and controlling time with a stopwatch, and counting the number of biceps curls (elbow flexion/extension with supination) was completed in 30 s. For the 30-second chair-stand test, participants were seated on a 40-cm-high chair with both legs shoulder-width apart and arms crossed in front of their chest. The participants were then instructed to stand and sit repeatedly for 30 s with their arms crossed. Similar to the arm curl test, the number of times a person completed the stand-to-sit sequence within 30 s was recorded [23]. In the present study, we measured handgrip strength as an indicator of whole-body isometric muscle strength. To measure handgrip strength, the participants stood upright and squeezed as hard as possible using a Smedley-type handgrip dynamometer (GRIPD; Takei, Niigata, Japan), with the grip size adjusted to a comfortable level.

### Gait speed

Gait speed (normal and maximum speed) was measured using a 5-m course. Time was recorded using a handheld stopwatch to the nearest 0.01 s.

### Body composition

Body composition (body fat percentage and skeletal muscle index [SMI]) was determined using the bioelectrical impedance method (InBody270, InBody Japan, Tokyo). Height and body weight were measured using standard diameters and scales. Body mass index (BMI) was determined by dividing body weight (kg) by the square of height (m^2^). Waist circumference was measured midway between the lowest rib and iliac crest at the end of gentle expiration while the subject was standing. The calf circumference was measured in a standing position at the point of maximum circumference on a plane perpendicular to the long axis of the calves, and the maximal value of both calves was used.

### Resting blood pressure

Systolic blood pressure (SBP) and diastolic blood pressure (DBP) were measured in the sitting position after 3 min of rest using an automatic sphygmomanometer (HCR-7501T; Omron, Kyoto, Japan).

### Food intake

To ease food intake assessment for older adults, food intake was assessed using a questionnaire on food intake frequency (Dietary Variety Scores; DVS) covering 1 week and covering the main 10 food groups in the Japanese diet. Before and after intervention, we evaluated the frequency of intake of 10 food groups (meat, fish/shellfish, eggs, milk, soybean products, green/yellow vegetables, potatoes, fruit, seaweed, and fats/oils, which constitute a large part of Japanese daily main and side dishes), developed by Kumagai et al. [24]. The DVS reflects the nutrient adequacy of the diet [25]. Participants were provided with a record sheet accompanied by illustrations of the 10 food groups. Based on the methodology by Kumagai et al. [24], they were instructed to check the box for each food group on any day they consumed it at least once during the 1-week period, regardless of the frequency of intake within a single day. The DVS was calculated as the mean number of the 10 predefined food groups consumed per day over one week (range: 0–10). For each day, participants received a score based on the number of food groups consumed, and the weekly average of these daily scores was used as the DVS. In addition, for each food group, the number of days on which the food group was consumed during the previous week was recorded (range: 0–7 days/week) and used to describe the consumption frequency of individual food groups.

### Daily logs (exercise and dairy consumption)

During the study, in the ME and exercise groups (both groups), exercise [≥ 3 times/week] (four exercises; shoulder and grip exercise using towels, knee extensions, and lunges) and dairy product consumption [daily] (milk supplement intake, dairy products intake excluding milk supplement and milk) adherence at home was measured using a self-reported log each day. The participants submitted their logs to the researchers once a month. Adherence rate during the intervention was calculated as follows: number of completed days/numbers of instructed days (exercise [≥ 3 times/week] and dairy product intake [daily]).

### Frailty

At baseline, physical frailty was assessed using the Japanese version of the Cardiovascular Health Study criteria (J-CHS), which has five components (shrinking, weakness, slowness, exhaustion, and low activity) and is based on the original criteria of the phenotype model proposed by Fried (2001) [26]. The criteria were optimized for older Japanese adults and validated elsewhere [27]. Frailty was defined as having three or more components, and pre-frailty was defined as having one or two components.

### Subgroup analysis based on baseline data (dairy consumption and SMI)

Subgroup analyses were pre-specified because dairy consumption and SMI were used as stratification variables during randomization and were considered potential effect modifiers. Many countries have issued guidelines recommending the intake of at least one serving of dairy products per day [28, 29]. According to this evidence, subgroup analyses were based on the baseline frequency of dairy consumption (High: ≥ 1 cup/day or Low: <1 cup/day) (according to the Japanese Food Guide Spinning Top) [17] and baseline SMI according to the definition of the Asia Sarcopenia Working Group) (AWGS2019; men < 7.0 kg/m^2^, women < 5.7 kg/m^2^) [30]. Participants with values above these cutoffs were defined as the high group, whereas the rest were defined as the low group.

### Statistical analysis

Comparisons of pre- and post-intervention values were performed using a repeated-measures two-way analysis of variance (group × time). The Shapiro-Wilk test was used to test for normality. Delta values, obtained by subtracting post-trial from pre-trial values, were used to estimate whether the changes in the parameters were clinically meaningful and examine the effect size. Differences in delta values were analyzed using the Mann–Whitney U test. The effect sizes (Cohen’s d) of the pre- and post-trial data were determined using average changes, excluding the pre-test standard deviation. The values were categorized as follows: small, 0.2 to < 0.5; moderate, 0.5 to < 0.8; large, ≥ 0.8. Within-group changes were analyzed using the paired t-test and Wilcoxon signed-rank test. Adherence outcomes were summarized using the median, interquartile range (IQRs), and range. Histograms were used to visualize adherence to the nutritional intervention, whereas resistance exercise adherence was illustrated using box plots with individual participant data points. Between-group differences in resistance exercise adherence were assessed using the Mann–Whitney U-test. Analyses were conducted using SPSS (ver. 28.0; IBM, New York, USA) and R (R Foundation for Statistical Computing, Vienna, Austria). Data are expressed as the mean ± standard deviation, and statistical significance was set at *P* < 0.05.

## Results

### Participants (Table 1)

**Table 1:** Baseline characteristics

		ME group (*n* = 27)		E group (*n* = 27)	
		Mean	SD	Mean	SD
	Sex (M/F)	male6 / female21		male7 / female20	
	Age (years)	77.6	6.9	79.1	6.7
Anthropometrics					
	Height (cm)	153.9	7.7	153.1	8.1
	Body weight (kg)	54.4	8.8	53.5	11.4
	BMI (kg/m^2^)	22.9	2.9	22.7	3.4
	Waist circumference (cm)	86.1	9.5	83.3	10.7
	Calf circumference (cm)	33.9	3.1	34.0	3.7
Resting blood pressure					
	Systolic blood pressure (mmHg)	136.2	14.6	141.7	14.5
	Diastolic blood pressure (mmHg)	79.9	8.9	84.8	9.6
Upper and lower limb strength					
	Arm curl test (reps)	18.4	4.7	16.9	3.9
	30-s chair stand test (reps)	15.6	4.2	14.6	3.5
Muscle strength					
	Handgrip strength (kg)	25.8	8.7	24.3	8.4
Gait speed					
	Normal speed (m/s)	1.3	0.3	1.3	0.3
	Maximum speed (m/s)	1.7	0.4	1.6	0.4
Body composition					
	Body fat (%)	31.5	7.0	29.2	6.8
	Skeletal muscle index (kg/m^2^)	6.1	1.1	6.3	1.2
Food intake					
	Dietary Variety Score (mean number of food groups/day) (0–10)	8.3	1.1	8.4	1.0
Frailty (J-CHS criteria)					
	Robust (*n*, %)	(11, 45.8%)		(13, 48.1%)	
	Prefrail (*n*, %)	(15, 55.6%)		(12, 44.4%)	
	Frail (*n*, %)	(1, 3.7%)		(2, 7.4%)	
Frailty components (J-CHS criteria)					
	Unintentional weight loss (*n*, %)	(11, 45.8%)		(4, 14.8%)	
	Low activity levels (*n*, %)	(6, 25.0%)		(8, 29.6%)	
	Exhaustion (*n*, %)	(2, 8.3%)		(5, 18.5%)	
	Weakness (*n*, %)	(3, 12.5%)		(5, 18.5%)	
	Slowness (*n*, %)	(4, 16.7%)		(2, 7.4%)	

Values are presented as the mean ± standard deviation

*ME* dairy products and resistance training, *E* resistance training, *J-CHS*Japanese version of the Cardiovascular Health Study

A total of 54 older adults (age ≥ 65 years) (men, *n* = 13; women, n = 41; mean age, 78.5 ± 6.7 years; BMI, 22.8 ± 3.1 kg/m^2^) were included in this study. During the study, three participants (ME group, *n* = 3; E group, *n* = 0) withdrew because of leg pain (*n* = 2) and constipation (*n* = 1). Finally, 51 participants (men, *n* = 13; women, *n* = 38; mean age, 78.2 ± 6.9 years, BMI, 22.9 ± 3.2 kg/m^2^) completed the study and were subsequently enrolled in the analysis (retention rate was 94.4%). There were no significant differences between the ME and E groups in age, sex distribution, or baseline DVS. Among the 54 eligible participants, three (5.6%) were classified as frail and 27 (50.0%) as pre-frail. The baseline characteristics were well balanced between the two groups (Table 1).

### Study adherence (dairy intake and home-based resistance training) (Supplementary Fig. 1a.b)

Adherence to milk supplement consumption was exceptionally high (median 7.00 times/week, IQR 7.00–7.00), while the median frequencies of milk and dairy product intakes were 6.83 (IQR 5.60–7.00) and 6.83 (IQR 6.17–6.94) times/week, respectively. Resistance exercise adherence did not differ between the ME and E groups (3.13 [2.81–3.65] vs. 3.25 [2.71–3.96] times/week (*p* = 0.940).

### Changes in parameters (Table 2, Supplementary Table 1)

**Table 2:** Changes after intervention

		ME group (*n*=24) (male *n*=6 / female *n*=18)						E group (*n*=27) (male *n*=7 / female *n*=20)										
		Pre		Post		Cohen's d	*p*-value *	Pre		Post		Cohen's d	*p*-value *	Two-way analysis of variance				
		Mean	SD	Mean	SD			Mean	SD	Mean	SD			Time		Group		Interaction
Anthropometrics																		
	Body weight (kg)	55.2	9.0	56.1	8.9	0.99	<0.001	53.5	11.4	53.6	11.1	0.19	0.341	<0.001		0.474		0.006
	BMI (kg/m^2^)	23.1	3.0	23.5	3.0	1.03	<0.001	22.7	3.4	22.7	3.3	0.21	0.276	<0.001		0.511		0.005
	Waist circumference (cm)	86.9	9.8	85.5	11.0	0.43	0.045	83.3	10.7	80.9	10.4	0.48	0.019	0.003		0.160		0.425
	Calf circumference (cm)	34.2	3.1	34.3	2.8	0.07	0.747	34.0	3.7	33.6	3.4	0.20	0.298	0.446		0.627		0.297
Resting blood pressure																		
	Systolic blood pressure (mmHg)	134.5	14.1	134.7	14.4	0.02	0.928	141.7	14.5	141.8	17.4	0.01	0.975	0.941		0.059		0.982
	Diastolic blood pressure (mmHg)	79.2	9.2	79.6	8.7	0.08	0.703	84.8	9.6	85.7	8.8	0.10	0.609	0.536		0.015		0.814
Upper and lower limb strength																		
	Arm curl test (reps)	18.8	4.6	20.1	4.0	0.46	0.033	16.9	3.9	18.0	4.8	0.35	0.084	0.006		0.083		0.797
	30-s chair stand test (reps)	15.8	4.2	16.4	4.0	0.36	0.090	14.6	3.5	15.6	2.5	0.46	0.025	0.006		0.308		0.395
Muscle strength																		
	Handgrip strength (kg)	26.9	8.5	26.0	7.7	0.50	0.022	24.3	8.4	24.2	7.7	0.02	0.915	0.115		0.332		0.156
Gait speed																		
	Normal speed (m/s)	1.3	0.3	1.2	0.2	0.89	<0.001	1.3	0.3	1.2	0.2	0.50	0.162	0.002		0.943		0.414
	Maximum speed (m/s)	1.7	0.4	1.6	0.3	0.48	0.033	1.6	0.4	1.6	0.3	0.38	0.116	0.007		0.681		0.510
Body composition																		
	Body fat (%)	30.8	7.1	32.7	7.7	0.62	0.006	29.2	6.8	29.9	6.2	0.25	0.199	0.002		0.257		0.120
	Skeletal muscle index (kg/m^2^)	6.3	1.0	6.2	1.0	0.53	0.016	6.3	1.2	6.0	1.1	1.00	<0.001	<0.001		0.762		0.134
Frailty																		
	Robust (*n*, %)	(11, 45.8%)						(13, 48.1%)										
	Prefrail (*n*, %)	(13, 54.2%)						(12, 44.4%)										
	Frail (*n*, %)	(0, 0%)						(2, 7.4%)										

Effect size (Cohen’s d): small, d = 0.2 to <0.5; moderate, d = 0.5 to <0.8; large, d ≥0.8

*Paired t-test

Across the entire study population, although significant time effects were found for walking speed and limb strength (upper and lower) (all *p* < 0.05), there was no interaction for any of the variables (Table 2). Food intake did not change before or after the trial in either group (ME or E group) (Supplementary Table 1). 

### Effects related to habitual dairy product intake (Tables 3 and 4, Fig. 3a-c, Supplementary Fig. 2a-c)

**Table 3:** Changes after intervention, among participants with low baseline dairy consumption (Low: <1 cup/day)

	ME-Low (*n*=12)						E-Low (*n*=14)						delta	delta
	Pre		Post		Cohen's d	*p*-value*	Pre		Post		Cohen's d	*p*-value*	Cohen's d	*p*-value **
	Mean	SD	Mean	SD			Mean	SD	Mean	SD				
Age (years)	75.4	5.9					77.1	6.2						
Anthropometrics														
Body weight (kg)	57.7	10.6	58.8	10.6	1.26	0.001	53.1	12.7	53.3	12.1	0.19	0.491	1.05	0.032
BMI (kg/m^2^)	23.7	3.3	24.1	3.3	1.22	0.001	22.2	3.6	22.3	3.3	0.24	0.382	1.00	0.042
Waist circumference (cm)	89.6	10.1	88.1	12.9	0.36	0.238	83.2	11.4	80.5	11.0	0.72	0.018	0.31	0.456
Calf circumference (cm)	34.6	2.9	34.8	2.9	0.38	0.215	33.8	4.0	33.5	3.8	0.26	0.345	0.60	0.141
Resting blood pressure														
Systolic blood pressure (mmHg)	135.5	16.4	135.6	17.0	0.01	0.982	143.9	16.6	141.2	18.2	0.16	0.567	0.18	1.000
Diastolic blood pressure (mmHg)	79.8	9.5	80.8	10.7	0.23	0.447	86.9	10.1	86.4	9.0	0.05	0.841	0.21	0.777
Upper and lower limb strength														
Arm curl test (reps)	17.7	3.6	19.9	3.1	0.98	0.006	18.5	3.0	18.8	4.9	0.08	0.771	0.64	0.223
30-s chair stand test (reps)	15.7	4.1	16.5	3.9	0.59	0.064	15.8	3.0	16.6	1.7	0.35	0.209	0.03	0.654
Muscle strength														
Handgrip strength (kg)	27.1	6.6	25.8	5.6	0.59	0.066	26.8	8.4	26.8	7.8	0.02	0.951	-0.62	0.157
Gait speed														
Normal speed (m/s)	1.4	0.3	1.2	0.2	0.89	0.010	1.4	0.3	1.3	0.2	0.36	0.205	0.13	0.520
Maximum speed (m/s)	1.7	0.3	1.7	0.3	0.21	0.480	1.7	0.4	1.7	0.3	0.18	0.503	0.07	0.487
Body composition														
Body fat (%)	31.9	6.9	34.3	7.5	0.79	0.020	28.2	5.9	29.5	5.7	0.60	0.042	0.74	0.085
Skeletal muscle index (kg/m^2^)	6.4	1.1	6.3	1.1	0.39	0.206	6.3	1.3	6.1	1.2	0.89	0.006	0.76	0.118

Effect size (Cohen’s d): small, d = 0.2 to <0.5; moderate, d = 0.5 to <0.8; large, d ≥0.8

*: Paired t-test

**: Mann–Whitney U test

**Table 4:** Changes after intervention, among participants with high baseline dairy consumption (High: ≥1 cup/day)

		ME-High (*n*=12)						E-High (*n*=13)						delta	delta
		Pre		Post		Cohen's d	*p*-value*	Pre		Post		Cohen's d	*p*-value*	Cohen's d	*p*-value **
		Mean	SD	Mean	SD			Mean	SD	Mean	SD				
	Age (years)	78.9	8.1					81.2	6.8						
Anthropometrics															
	Body weight (kg)	52.7	6.6	53.3	6.1	0.76	0.023	53.9	10.4	54.1	10.5	0.18	0.538	0.56	0.275
	BMI (kg/m^2^)	22.5	2.6	22.8	2.7	0.86	0.013	23.1	3.3	23.1	3.3	0.18	0.541	0.62	0.221
	Waist circumference (cm)	84.3	9.0	82.9	8.6	-0.58	0.069	83.4	10.5	81.4	10.0	-0.33	0.260	0.14	0.935
	Calf circumference (cm)	33.9	3.3	33.8	2.7	-0.12	0.688	34.2	3.5	33.7	2.9	-0.20	0.481	0.19	0.586
Resting blood pressure															
	Systolic blood pressure (mmHg)	133.5	12.0	133.8	12.0	0.03	0.914	139.4	12.0	142.5	17.3	0.16	0.579	-0.17	0.586
	Diastolic blood pressure (mmHg)	78.7	9.3	78.4	6.2	-0.04	0.886	82.5	8.8	84.9	8.9	0.26	0.373	-0.34	0.276
Upper and lower limb strength															
	Arm curl test (reps)	19.8	5.3	20.3	4.9	0.13	0.661	15.1	4.1	17.1	4.6	0.77	0.016	-0.55	0.441
	30-s chair stand test (reps)	15.9	4.4	16.3	4.2	0.18	0.540	13.2	3.7	14.6	2.9	0.55	0.072	-0.47	0.202
Muscle strength															
	Handgrip strength (kg)	26.8	10.4	26.2	9.6	-0.39	0.207	21.6	7.9	21.5	6.7	-0.05	0.858	-0.19	0.399
Gait speed															
	Normal speed (m/s)	1.3	0.4	1.2	0.3	-0.87	0.012	1.3	0.3	1.1	0.2	-0.65	0.037	0.09	0.978
	Maximum speed (m/s)	1.7	0.4	1.5	0.3	-0.80	0.018	1.6	0.3	1.5	0.3	-0.80	0.014	0.31	0.221
Body composition															
	Body fat (%)	29.7	7.5	31.1	7.9	0.44	0.154	30.3	7.7	30.3	6.9	0.01	0.978	0.62	0.320
	Skeletal muscle index (kg/m^2^)	6.2	1.0	6.0	0.9	-0.66	0.042	6.2	1.1	6.0	1.1	-1.13	0.002	0.14	0.979

*: Paired t-test

**: Mann–Whitney U test

**Fig. 3:**
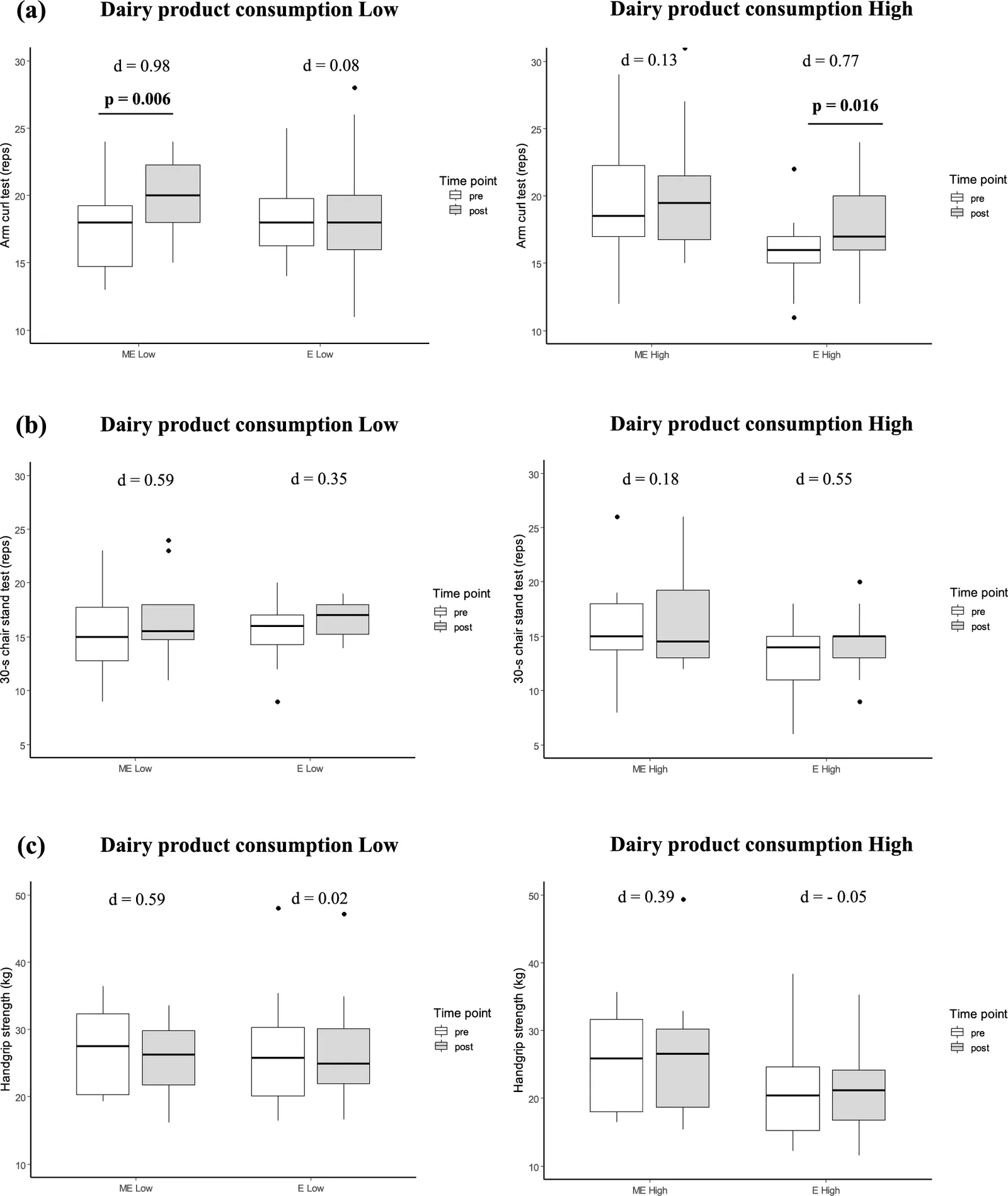
**a** Changes in upper limb strength by group at baseline and follow-up, stratified according to baseline dairy product consumption. ME, dairy product and resistance training group; E, resistance training group; High, baseline frequency of dairy product consumption ≥ 1 cup/day; Low, baseline frequency of dairy product consumption < 1 cup/day. Effect size (Cohen’s d): small, d = 0.2–<0.5; moderate, d = 0.5 < 0.8; large, d ≥ 0.8. **b** Changes in lower limb strength by group at baseline and follow-up, stratified according to baseline dairy products consumption. **c** Changes in handgrip strength by group at baseline and follow-up, stratified according to baseline dairy products consumption

A possible threshold for the effect of dairy products on physical function in older adults has been suggested for seven or more servings of dairy products per week [14]. At baseline, half of the participants in both the ME and E groups exceeded this threshold. In a post hoc analysis (Tables 3 and 4), participants in each group were divided into two subgroups based on their baseline daily dairy product consumption: those consuming less than 1 cup/day (low) and those consuming ≥ 1 cup/day (high). The effects of the interventions were analyzed in these subgroups. In the ME group, participants with low baseline dairy product consumption (ME-Low) showed significant improvements in upper-limb muscle strength (*p* = 0.006) and a trend toward improvement in lower-limb muscle strength (*p* = 0.064). Specifically, upper limb muscle strength increased from 17.7 ± 3.6 reps at baseline to 19.9 ± 3.1 reps after 3 months in the ME-Low group, while it remained essentially unchanged in the E-Low group (18.5 ± 3.0 reps at baseline and 18.8 ± 4.9 reps at 3 months). The effect size for the ME-Low group was large (d = 0.98), whereas that for the E-Low group was negligible (d = 0.08). Similarly, in the ME-Low group, lower limb muscle strength improved from 15.7 ± 4.1 reps to 16.5 ± 3.9 reps (d = 0.59; moderate), whereas in the E-Low group, it changed from 15.8 ± 3.0 reps to 16.6 ± 1.7 reps (d = 0.35; small) (Fig. 3a-c, Supplementary Table 2a) No significant effects related to dairy intake level were observed for resting blood pressure or muscle mass. In contrast, when stratified by baseline muscle mass (high vs. low SMI), the intervention effects were minimal and did not reach statistical significance. Specifically, the effect sizes for upper and lower limb strength in the ME–SMI low group were negligible (d < 0.04) (Supplementary Table 2b, Supplementary Fig. 2a-c).

## Discussion

In this pragmatic RCT, we investigated whether adding modest daily dairy product intake to home-based resistance training improves physical function in community-dwelling older adults in a real-world setting. The data showed that limb strength improved over time, irrespective of group assignment, and between-group differences were not statistically significant. This indicates that the combination of dairy *product* intake and exercise did not produce superior effects to exercise alone. However, in the exploratory subgroup analyses, participants with lower habitual dairy intake in ME group exhibited improvements in limb strength when modest dairy consumption was added to their training regimen. These results suggest that baseline nutritional status, particularly habitual dairy consumption, may modify responsiveness to combined nutrition‑and‑exercise interventions. Future research should explore the mechanisms underlying these effects, and determine the optimal amount of dairy intake and resistance training required.

Although no significant differences in upper and lower limb performance were observed between the ME and E groups, improvements in both upper (arm curl test) (*p* = 0.033) and lower (30-s chair stand test) (*p* = 0.090) limb strengths were noted only in the ME group (Table 2). Notably, these improvements were limited to participants with lower habitual dairy consumption at baseline (the ME-Low subgroup). These findings are consistent with those of previous studies. For instance, Al-Rawhani et al. reported that whey protein supplementation enhanced lower body strength, but did not significantly improve handgrip strength or body composition in older adults [31]. Although not specifically examining dairy consumption, Campbell and Leidy reported that protein supplementation increased muscle mass and strength primarily among older adults whose habitual protein intake was below the recommended dietary allowance (< 0.8 g· kg/d), suggesting that baseline nutritional status may influence responsiveness to nutritional interventions [32]. Taken together with previous evidence showing attenuated responses among individuals with adequate baseline nutrient intake, our findings support the possibility that baseline dietary habits may influence the responsiveness to combined nutrition and exercise interventions.

Our observation that baseline dietary intake influences intervention outcomes is supported by previous studies, particularly those regarding body composition among middle-aged obese individuals. Wennersberg et al. conducted a six-month milk intake study and found no significant differences between the intervention and control groups [13]. However, a subgroup analysis revealed that participants with low baseline calcium intake (< 700 mg/day) experienced significant reductions in waist circumference and abdominal fat [13]. Milk and milk products are rich in calcium [1]. Although the outcomes differed from those of the present study, these findings suggest that individuals with poorer baseline nutritional status may derive greater benefits from dairy product interventions than those with adequate nutrient intake. Similarly, Verdijk et al. reported that protein supplementation before and after exercise did not increase the skeletal muscle mass or strength in older men who habitually consumed adequate amounts of dietary protein [11]. Interestingly, Zhang et al. also found that the benefits of protein supplementation on muscle health were limited to older individuals with poor lifestyle habits such as physical inactivity [33]. Taken together, these findings suggest that individuals with poor baseline nutritional status may experience greater health benefits from interventions that closely resemble their usual lifestyles.

Although body fat increased and gait speed decreased in some analyses, these changes cannot be attributed to dairy product intake because the study was not designed to determine causality. The modest amount of protein provided (10–15 g/day) and unsupervised, low-intensity exercise might also have been below the threshold needed to stimulate muscle protein synthesis in older adults. Previous studies have similarly suggested that a higher protein intake or more structured training may be required to produce measurable improvements. Therefore, future research should examine appropriate protein dosage and training intensity while taking habitual dairy intake into account. Churchward-Venne et al. suggested that higher protein supplementation (~ 40 g/day) combined with resistance training might be more effective in increasing skeletal muscle mass [34]. Future studies should consider both habitual dairy intake and the optimal protein dosage.

The present study had several strengths and limitations. The strengths include controlled and randomized design and real-world experimental settings. Thus, the findings of the present study may fairly mimic the behavior of the general elderly population in their daily lives. This type of dietary intervention, in which no instructions are provided regarding energy restriction or other dietary changes, giving it high external validity, is currently rare and needed. The limitations included a small sample size, a relatively short intervention period (three months), and the fact that the use of a dietary intake assessment was not validated. Therefore, misclassification of habitual dairy consumption and intervention adherence may have occurred, particularly in subgroup analyses based on baseline dairy consumption. However, because participants were categorized using a simple frequency-based threshold (≥ 1 cup/day vs. <1 cup/day) derived from the Japanese Food Guide Spinning Top [17], we believe that our subgroup classification is reasonably accurate. Second, our participants might have been somewhat healthier and more health-conscious than the general elderly population. For instance, the frailty prevalence in our participants (5.6%) was slightly lower than that in older Japanese adults (8.7%) [35]. Moreover, our participants’ nutrition-related health consciousness appeared to be higher than that of the general older adults. In fact, the DVS (out of 10 points) in our participants (8.3 ± 1.1 in ME and 8.4 ± 1.0 in E) was greater than the highest tertile in DVS among Chinese older adults (4.4–8.0 points, out of 8 points) [36]. The relatively high dietary quality and frequent dairy consumption observed in our participants may have reduced the likelihood of detecting additional benefits of dairy intake, which could partly explain the absence of significant between-group effects. Third, dairy intake was assessed qualitatively via self-reported frequency logs rather than quantitative dietary records; therefore, it could be overestimated. Nevertheless, based on the participants’ logs, most participants consumed the assigned volume of dairy products (milk, milk products, and milk supplements) (Supplementary Fig. 1a). Given that the amount of protein in the standard dairy products was one glass of milk (200 mL) (approximately 6.8 g of protein), one cup of yogurt (approximately 4.5 to 5.0 g of protein) [37], and one stick of milk supplement (3.8 g of protein), the total protein consumed by the participants during the study corresponded to our targeted protein amount (11–15 g/day). Future trials should include validated dietary assessment tools or biomarkers to objectively estimate dairy protein intake and compliance. Fourth, the absence of blinding in this study may have introduced systematic bias, particularly in self-reported outcomes such as exercise frequency and dairy product intake. To address these issues, future studies should incorporate objective measures of dietary intake, such as weighed food records, in all study groups.

## Conclusions

Under real-world conditions, the addition of modest dairy product intake to home-based resistance training did not enhance overall physical function compared with resistance training alone in community-dwelling older adults. Nevertheless, improvements in upper- and lower-limb strength were observed among participants with low habitual dairy consumption at baseline, whereas no consistent effects were observed among those with higher baseline intake or when stratified by baseline muscle mass. These findings suggest that baseline dietary habits may modify the response to combined nutrition and exercise interventions. However, given the absence of significant between-group differences and the exploratory nature of the subgroup analyses, the results should be interpreted cautiously. Further adequately powered trials using precise dietary assessment methods are needed to clarify the potential role of dairy intake in supporting the physical function of older adults.

## Supplementary Information

Supplementary Material 1.

Supplementary Material 2.

## Data Availability

The datasets used and/or analyzed during the present study are available from the corresponding author upon reasonable request.
