# Supplementary material for: Effects of dairy product intake and home-based resistance exercise on the physical function in Japanese community-dwelling older adults: results of a real-world randomized controlled trial
Source: BMC Geriatr. 2026 Aug 4;26:1370. doi: 10.1186/s12877-026-08061-x (PMC13721421; doi:10.1186/s12877-026-08061-x)
Supplement: Supplementary file 1 [file 12877_2026_8061_MOESM2_ESM.docx]

**Supplementary files**

| Supplementary Table 1. Changes in food intake | | | | | | | | | | | | | | | | |
| --- | --- | --- | --- | --- | --- | --- | --- | --- | --- | --- | --- | --- | --- | --- | --- | --- |
|  | | | | | | | | | | | | | | | | |
|  |  | ME group (n=24) (male n=6 / female n=18) | | | | | | |  | E group (n=27) (male n=7 / female n=20) | | | | | | |
|  |  | Pre | |  | Post | |  | p-value ^$^ |  | Pre | |  | Post | |  | p-value ^$^ |
|  |  | Mean | SD |  | Mean | SD |  |  |  | Mean | SD |  | Mean | SD |  |  |
| Dietary Variety Score (mean number of food groups/day) (0-10) | | 8.4 | 0.9 |  | 8.2 | 1.0 |  | 0.175 |  | 8.4 | 1.0 |  | 8.4 | 1.0 |  | 0.830 |
| Consumption frequency of individual food groups (days/week) (0-7) | | | | | | | | | | | | | | | | |
|  | Meat | 5.7 | 1.6 |  | 5.8 | 1.7 |  | 0.655 |  | 5.8 | 1.3 |  | 5.8 | 1.2 |  | 0.439 |
|  | Eggs | 6.0 | 1.5 |  | 5.7 | 1.5 |  | 0.283 |  | 6.1 | 1.2 |  | 6.0 | 1.1 |  | 0.719 |
|  | Milk | 6.6 | 1.3 |  | 6.3 | 1.6 |  | 0.303 |  | 6.5 | 1.4 |  | 6.8 | 0.4 |  | 0.558 |
|  | Fats and Oils | 6.2 | 1.7 |  | 5.5 | 1.8 |  | 0.054 |  | 6.3 | 1.6 |  | 6.0 | 1.3 |  | 0.127 |
|  | Seafood | 5.3 | 1.6 |  | 5.3 | 1.7 |  | 0.538 |  | 5.4 | 1.2 |  | 5.2 | 1.4 |  | 0.419 |
|  | Soybeans and Soy Products | 5.8 | 1.2 |  | 6.1 | 1.4 |  | 0.167 |  | 6.3 | 1.0 |  | 6.0 | 1.2 |  | 0.134 |
|  | Green and Yellow Vegetables | 6.7 | 0.7 |  | 6.6 | 1.3 |  | 0.914 |  | 6.7 | 0.9 |  | 6.7 | 0.7 |  | 0.680 |
|  | Potatoes | 4.6 | 2.1 |  | 4.5 | 1.8 |  | 0.701 |  | 4.2 | 1.9 |  | 4.6 | 2.2 |  | 0.271 |
|  | Fruits | 6.5 | 1.1 |  | 6.8 | 0.5 |  | 0.161 |  | 6.6 | 1.0 |  | 6.7 | 0.7 |  | 0.803 |
|  | Seaweed | 5.4 | 1.6 |  | 4.4 | 2.2 |  | **0.033** |  | 5.2 | 1.4 |  | 5.2 | 1.3 |  | 0.979 |
| $: Wilcoxon signed-rank test | | | | | | | | | | | | | | | | |
| The Dietary Variety Score (DVS) was calculated as the average number of food groups consumed per day over one week. For each day, participants received a score based on the number of the 10 predefined food groups consumed (range: 0–10). | | | | | | | | | | | | | | | | |

| Supplementary Table 2a. Changes after intervention, among participants with low baseline skeletal muscle mass index (SMI-Low) (<7.0kg/m^2^ for men and <5.7 kg/m^2^ for women). | | | | | | | | | | | | | | | | | | | | | | |
| --- | --- | --- | --- | --- | --- | --- | --- | --- | --- | --- | --- | --- | --- | --- | --- | --- | --- | --- | --- | --- | --- | --- |
|  | | | | | | | | | | | | | | | | | | | | | | |
|  |  | ME-SMI Low (n=7) | | | | | | | |  |  | E-SMI Low (n=10) | | | | | | | |  | delta | delta |
|  |  | Pre | |  | Post | |  | Cohen's d | p-value* |  |  | Pre | |  | Post | |  | Cohen's d | p-value* |  | Cohen's d | p-value ** |
|  |  | Mean | SD |  | Mean | SD |  |  |  |  |  | Mean | SD |  | Mean | SD |  |  |  |  |  |  |
|  | Age (years) | 79.4 | 9.2 |  |  |  |  |  |  |  |  | 83.1 | 5.8 |  |  |  |  |  |  |  |  |  |
| Anthropometrics | |  |  |  |  |  |  |  |  |  |  |  |  |  |  |  |  |  |  |  |  |  |
|  | Body weight (kg) | 47.5 | 4.0 |  | 48.5 | 4.2 |  | 1.13 | **0.024** |  |  | 43.1 | 5.3 |  | 43.4 | 5.2 |  | 0.32 | 0.340 |  | 0.78 | 0.133 |
|  | BMI (kg/m^2^) | 20.6 | 1.9 |  | 21.0 | 2.1 |  | 1.07 | **0.030** |  |  | 19.7 | 2.2 |  | 19.9 | 2.3 |  | 0.36 | 0.287 |  | 0.71 | 0.174 |
|  | Waist circumference (cm) | 82.8 | 6.4 |  | 78.9 | 8.6 |  | 1.30 | **0.014** |  |  | 74.2 | 6.7 |  | 72.8 | 7.0 |  | 0.29 | 0.385 |  | 0.65 | 0.178 |
|  | Calf circumference (cm) | 31.5 | 2.1 |  | 31.9 | 2.4 |  | 0.84 | 0.069 |  |  | 30.6 | 2.0 |  | 31.0 | 2.1 |  | 0.34 | 0.311 |  | 0.04 | 0.933 |
| Resting blood pressure | |  |  |  |  |  |  |  |  |  |  |  |  |  |  |  |  |  |  |  |  |  |
|  | Systolic blood pressure (mmHg) | 131.9 | 13.9 |  | 130.4 | 13.6 |  | 0.39 | 0.340 |  |  | 145.8 | 15.6 |  | 136.1 | 11.2 |  | 0.50 | 0.149 |  | 0.54 | 0.219 |
|  | Diastolic blood pressure (mmHg) | 76.9 | 7.8 |  | 76.1 | 10.5 |  | 0.20 | 0.622 |  |  | 88.1 | 12.6 |  | 84.3 | 8.2 |  | 0.37 | 0.276 |  | 0.37 | 0.402 |
| Upper and lower limb strength | |  |  |  |  |  |  |  |  |  |  |  |  |  |  |  |  |  |  |  |  |  |
|  | Arm curl test (reps) | 17.6 | 3.2 |  | 18.9 | 3.5 |  | 0.39 | 0.336 |  |  | 15.2 | 4.4 |  | 15.2 | 4.1 |  | 0.00 | 1.000 |  | 0.47 | 0.389 |
|  | 30-s chair stand test (reps) | 15.9 | 3.3 |  | 15.7 | 3.8 |  | 0.21 | 0.604 |  |  | 14.1 | 3.8 |  | 15.2 | 2.9 |  | 0.69 | 0.057 |  | 0.95 | **0.047** |
| Muscle strength | |  |  |  |  |  |  |  |  |  |  |  |  |  |  |  |  |  |  |  |  |  |
|  | Handgrip strength (kg) | 22.1 | 5.4 |  | 21.0 | 5.6 |  | 0.62 | 0.153 |  |  | 18.2 | 4.5 |  | 18.5 | 4.1 |  | 0.15 | 0.653 |  | 0.65 | 0.180 |
| Gait speed | |  |  |  |  |  |  |  |  |  |  |  |  |  |  |  |  |  |  |  |  |  |
|  | Normal (m/s) | 1.3 | 0.4 |  | 1.2 | 0.3 |  | 0.98 | **0.041** |  |  | 1.3 | 0.4 |  | 1.2 | 0.3 |  | 0.37 | 0.275 |  | 0.35 | 0.457 |
|  | Maximum (m/s) | 1.7 | 0.4 |  | 1.5 | 0.3 |  | 1.11 | **0.026** |  |  | 1.5 | 0.3 |  | 1.4 | 0.3 |  | 0.38 | 0.258 |  | 0.96 | 0.094 |
| Body composition | |  |  |  |  |  |  |  |  |  |  |  |  |  |  |  |  |  |  |  |  |  |
|  | Body fat (%） | 32.5 | 3.4 |  | 32.4 | 4.0 |  | 0.14 | 0.726 |  |  | 31.0 | 2.8 |  | 30.9 | 3.0 |  | 0.07 | 0.829 |  | 0.22 | 0.627 |
|  | Skeletal muscle index (kg/m^2^) | 5.3 | 0.6 |  | 5.3 | 0.7 |  | 0.29 | 0.476 |  |  | 5.2 | 0.4 |  | 5.0 | 0.3 |  | 0.79 | **0.035** |  | 0.54 | 0.286 |
| Effect size (Cohen’s d): small, d = 0.2 to <0.5; moderate, d = 0.5 to <0.8; large, d ≥0.8. | | | | | | | | | | | | | | | | | | | | | | |
| *: Paired t-test | | | | | | | | | | | | | | | | | | | | | | |
| **: Mann–Whitney U test | | | | | | | | | | | | | | | | | | | | | | |

| Supplementary Table2b. Changes after intervention, among participants with high baseline skeletal muscle mass index (SMI-High) (≥7.0kg/m^2^ for men and ≥5.7 kg/m^2^ for women) | | | | | | | | | | | | | | | | | | | | | |
| --- | --- | --- | --- | --- | --- | --- | --- | --- | --- | --- | --- | --- | --- | --- | --- | --- | --- | --- | --- | --- | --- |
|  | | | | | | | | | | | | | | | | | | | | | |
|  |  | ME-SMI High (n=17) | | | | | | | |  | E-SMI High (n=17) | | | | | | | |  | delta | delta |
|  |  | Pre | |  | Post | |  | Cohen's d | p-value* |  | Pre | |  | Post | |  | Cohen's d | p-value* |  | Cohen's d | p-value ** |
|  |  | Mean | SD |  | Mean | SD |  |  |  |  | Mean | SD |  | Mean | SD |  |  |  |  |  |  |
|  | Age (years) | 76.2 | 6.2 |  |  |  |  |  |  |  | 76.7 | 6.2 |  |  |  |  |  |  |  |  |  |
| Anthropometrics | |  |  |  |  |  |  |  |  |  |  |  |  |  |  |  |  |  |  |  |  |
|  | Body weight (kg) | 58.4 | 8.6 |  | 59.2 | 8.5 |  | 0.91 | **0.002** |  | 59.6 | 9.5 |  | 59.7 | 9.1 |  | 0.10 | 0.696 |  | 0.78 | 0.133 |
|  | BMI (kg/m^2^) | 24.1 | 2.7 |  | 24.5 | 2.8 |  | 1.00 | **0.001** |  | 24.4 | 2.7 |  | 24.4 | 2.6 |  | 0.11 | 0.666 |  | 0.71 | 0.174 |
|  | Waist circumference (cm) | 88.6 | 10.5 |  | 88.3 | 11.0 |  | 0.13 | 0.593 |  | 88.7 | 8.9 |  | 85.7 | 9.0 |  | 0.58 | **0.030** |  | 0.65 | 0.178 |
|  | Calf circumference (cm) | 35.3 | 2.7 |  | 35.2 | 2.4 |  | 0.11 | 0.665 |  | 36.0 | 3.0 |  | 35.2 | 3.0 |  | 0.42 | 0.103 |  | 0.04 | 0.933 |
| Resting blood pressure | |  |  |  |  |  |  |  |  |  |  |  |  |  |  |  |  |  |  |  |  |
|  | Systolic blood pressure (mmHg) | 135.6 | 14.4 |  | 136.5 | 14.8 |  | 0.07 | 0.784 |  | 139.3 | 13.7 |  | 145.2 | 19.8 |  | 0.40 | 0.122 |  | 0.54 | 0.219 |
|  | Diastolic blood pressure (mmHg) | 80.2 | 9.8 |  | 81.1 | 7.7 |  | 0.15 | 0.544 |  | 82.8 | 7.1 |  | 86.5 | 9.3 |  | 0.49 | 0.061 |  | 0.37 | 0.402 |
| Upper and lower limb strength | |  |  |  |  |  |  |  |  |  |  |  |  |  |  |  |  |  |  |  |  |
|  | Arm curl test (reps) | 19.2 | 5.1 |  | 20.6 | 4.2 |  | 0.48 | 0.066 |  | 17.8 | 3.3 |  | 19.6 | 4.5 |  | 0.50 | 0.057 |  | 0.47 | 0.389 |
|  | 30-s chair stand test (reps) | 15.8 | 4.6 |  | 16.7 | 4.1 |  | 0.49 | 0.060 |  | 14.8 | 3.5 |  | 15.9 | 2.3 |  | 0.39 | 0.132 |  | 0.95 | 0.047 |
| Muscle strength | |  |  |  |  |  |  |  |  |  |  |  |  |  |  |  |  |  |  |  |  |
|  | Handgrip strength (kg) | 28.9 | 8.9 |  | 28.0 | 7.7 |  | 0.45 | 0.083 |  | 27.9 | 8.2 |  | 27.6 | 7.3 |  | 0.12 | 0.641 |  | 0.65 | 0.180 |
| Gait speed | |  |  |  |  |  |  |  |  |  |  |  |  |  |  |  |  |  |  |  |  |
|  | Normal (m/s) | 1.3 | 0.3 |  | 1.2 | 0.2 |  | 0.83 | **0.003** |  | 1.4 | 0.3 |  | 1.3 | 0.2 |  | 0.58 | **0.030** |  | 0.35 | 0.457 |
|  | Maximum (m/s) | 1.7 | 0.3 |  | 1.6 | 0.3 |  | 0.30 | 0.235 |  | 1.7 | 0.4 |  | 1.7 | 0.3 |  | 0.37 | 0.144 |  | 0.96 | 0.094 |
| Body composition | |  |  |  |  |  |  |  |  |  |  |  |  |  |  |  |  |  |  |  |  |
|  | Body fat (kg） | 17.8 | 5.4 |  | 19.2 | 5.5 |  | 0.74 | **0.007** |  | 17.9 | 4.3 |  | 18.3 | 4.3 |  | 0.39 | 0.131 |  | 0.73 | **0.045** |
|  | Body fat (%） | 40.3 | 7.7 |  | 39.7 | 7.9 |  | 0.31 | 0.225 |  | 41.4 | 8.2 |  | 41.1 | 7.8 |  | 0.24 | 0.345 |  | 0.56 | 0.118 |
|  | Skeletal muscle index (kg/m^2^) | 6.7 | 0.9 |  | 6.5 | 0.9 |  | 0.62 | **0.021** |  | 6.9 | 1.1 |  | 6.6 | 1.0 |  | 1.12 | **<0.001** |  | 0.42 | 0.235 |
| *: Paired t-test | | | | | | | | | | | | | | | | | | | | | |
| **: Mann–Whitney U test | | | | | | | | | | | | | | | | | | | | | |

Supplementary Figure 1a. Adherence (times/week) to dairy product intake in the ME group. (A) milk supplement, (B) milk intake, and (C) dairy product intake excluding the milk supplement. Dashed vertical lines indicate median values.

Supplementary Figure 1b. Adherence (times/week) to resistance exercise in the ME and E groups. Boxplots show the median, interquartile range, and range, while individual points represent participant-level data. Resistance exercise adherence was comparable between groups (p = 0.940).

Supplementary Fig. 2a. Changes in upper limb strength at baseline and after the intervention, stratified according to baseline skeletal muscle mass index.

ME, dairy product and resistance training group; E, resistance training group; SMI High, ≥7.0 kg/m^2^ for men and ≥5.7 kg/m^2^ for women; SMI Low, <7.0 kg/m^2^ for men and <5.7 kg/m^2^ for women.

Supplementary Fig. 2b. Changes in lower limb strength at baseline and after the intervention, stratified according to baseline skeletal muscle index.

Supplementary Fig. 2c. Changes in handgrip strength at baseline and after the intervention, stratified according to baseline skeletal muscle mass index.
